# Impact of reliable light and electricity on job satisfaction among maternity health workers in Uganda: A cluster randomized trial

**DOI:** 10.1186/s12960-022-00722-3

**Published:** 2022-03-29

**Authors:** Wei Chang, Jessica Cohen, Brian Mwesigwa, Peter Waiswa, Slawa Rokicki

**Affiliations:** 1 Harvard T.H. Chan School of Public Health, 90 Smith St, 3rd Floor, Boston, MA 02120 United States of America; 2 Innovations for Poverty Action, Kampala, Uganda; 3 School of Public Health, Makerere University, Kampala, Uganda; 4 Rutgers School of Public Health, Piscataway, United States of America

**Keywords:** Job satisfaction, Maternal and newborn health, Solar energy, Electricity and light, Mixed-methods, Uganda

## Abstract

**Background:**

Maintaining a motivated health workforce is critical to health system effectiveness and quality of care. Scant evidence exists on whether interventions aimed to strengthen health infrastructure in low-resource settings affect health workers. This study evaluated the impact of an intervention providing solar light and electricity to rural maternity facilities in Uganda on health workers’ job satisfaction.

**Methods:**

We used a mixed-methods design embedded in a cluster randomized trial to evaluate whether and how the We Care Solar Suitcase intervention, a solar electric system providing lighting and power, affected health workers in rural Ugandan maternity facilities with unreliable light. Facilities were randomly assigned to receive the intervention or not without blinding in a cluster-randomized controlled trial. Outcomes were assessed through two rounds of surveys with health workers. We used regression analyses to examine the intervention’s impact on job satisfaction. We used an inductive approach to analyze qualitative data to understand the study context and interpret quantitative findings.

**Results:**

We interviewed 85 health workers across 30 facilities, the majority of whom were midwives or nurses. Qualitative reports indicated that unreliable light made it difficult to provide care, worsened facility conditions, and harmed health workers and patients. Before the intervention, only 4% of health workers were satisfied with their access to light and electricity. After the installation, satisfaction with light increased by 76 percentage points [95% confidence interval (CI): 61–92 percentage points], although satisfaction with electricity did not change. Experience of negative impacts of lack of overhead light also significantly decreased and the intervention modestly increased job satisfaction. Qualitative evidence illustrated how the intervention may have strengthened health workers’ sense of job security and confidence in providing high-quality care while pointing towards implementation challenges and other barriers health workers faced.

**Conclusions:**

Reliable access to light and electricity directly affects health workers’ ability to provide maternal and neonatal care and modestly improves job satisfaction. Policy makers should invest in health infrastructure as part of multifaceted policy strategies to strengthen human resources for health and to improve maternal and newborn health services.

*Trial registration* socialscienceregistry.org: AEARCTR-0003078. Registered June 12, 2018, https://www.socialscienceregistry.org/trials/3078

Additionally registered on: ClinicalTrials.gov: NCT03589625, Registered July 18, 2018, https://clinicaltrials.gov/ct2/show/NCT03589625)

## Background

Improving maternal and newborn care quality is critical for reaching the Sustainable Development Goals and achieving universal health coverage. Sub-Saharan Africa (SSA) has the highest burden of maternal and perinatal mortality, accounting for 66% of maternal deaths and 42% of neonatal deaths globally [1, 2]. Most of these adverse outcomes can be prevented with high quality care [1], but many health facilities in SSA fall short of care that is “safe, effective, timely, efficient, equitable, and people-centered” as recommended by the World Health Organization [3, 4].

The health care workforce is the most important resource for health systems to provide high quality care and a critical building block to meet countries’ health goals [5, 6]. SSA has only 12.8 skilled health workers per 10,000 population, compared to the global average of 52.8 [7]. For health systems to tackle the health workforce shortage and to provide high quality of care, it is a priority to maintain a motivated health care workforce [8, 9]. This is of particular importance for maternity care workers in SSA, who provide essential services around the clock and often in remote rural areas with inadequate health infrastructure. However, levels of job satisfaction and morale are generally low as less than half of midwives and others who provided maternity care reported being satisfied with their current job in previous studies in SSA [10–12].

Health infrastructure, including access to electricity and light in health facilities, is critical to maternal health care delivery. According to a study across eight countries in SSA, 28% of health facilities are not connected to the power grid and 34% of hospitals suffer from frequent power outages [13]. Existing evidence suggests that lack of reliable light affects health workers’ job performance, job satisfaction, morale, and retention [14–20]. Some health workers in Kenya, Nigeria, and Tanzania identified inadequate access to electivity as a critical factor affecting their motivation and retention [14, 17, 20]. Maternity health workers in Burkina Faso and Tanzania prefer to serve in urban over rural areas partially due to availability of electricity [18]. Recent systematic reviews have associated poor physical work conditions with high levels of health worker burnout [21, 22]. A study in Uganda found that poor infrastructure at maternity facilities frustrated health workers and made them feel they could not offer quality care to patients [23]. In Tanzania, facility infrastructure and supportive interpersonal relationships were highly correlated with providers’ job satisfaction [24]. Electricity and light are particularly important to health workers in rural areas, where power grid access is limited and health workers typically use accommodations provided by health facilities [19, 20, 23]. However, there is little empirical evidence as to whether interventions aimed to strengthen health infrastructure can improve health workers’ job satisfaction.

We conducted a cluster-randomized controlled trial to evaluate the impact of the “We Care Solar Suitcase,” a solar electric system that provides medical lighting and electricity, on health workers’ job satisfaction in maternity facilities in rural Uganda. Using a mixed-methods approach, this study examines whether and how access to light and electricity affects health workers’ outcomes.

## Methods

### Study setting

The study was conducted in maternity facilities in Central, Eastern, and Western regions of Uganda [25]. Uganda has a population of 44.3 million in 2019, with a total fertility rate of 4.8 live births per woman [26]. In 2016, about 74.2% of deliveries were assisted by a skilled birth attendant and 73.4% of deliveries occurred in a health facility [27]. Uganda’s health system is divided into public and private sectors. The public sector consists of national and regional hospitals and a tiered district health system composed of health centers at four levels. Most lower-level health centers are not connected to the central electricity grid and power shortages are frequent [28, 29]. A 2015 survey suggested that kerosene lamps supplied 42% of lighting needs in Health Center IIs [29]. In our study, 40% of health facilities did not have electricity or relied on lanterns as the primary source of light at the time of the baseline survey [30].

### Intervention

The intervention, a “Solar Suitcase” manufactured by the non-government organization We Care Solar, is a complete solar electric system that contains high efficiency movable LED lights for medical use, rechargeable headlamps, USB ports for charging cell phones and small medical devices, and a portable fetal heart rate Doppler [30]. One Solar Suitcase was installed in each facility, with 2–4 overhead LED lights for each delivery room, depending on its size. A full description of the Solar Suitcase and its implementation, including training of health workers, maintenance, and costs have been previously published [25, 30].

### Study design

We used an embedded mixed-methods design to evaluate whether and how the intervention affected health workers’ job satisfaction and their experiences of working at night [31, 32]. Specifically, we embedded qualitative data collection within an experimental design before and after the intervention implementation to understand the study context and interpret quantitative results.

**Fig. 1:**
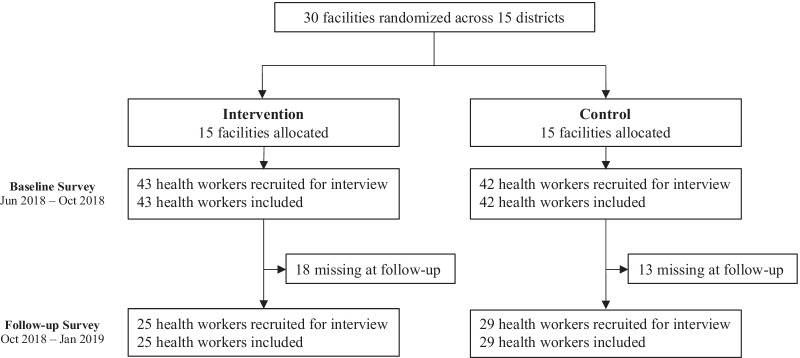
Study flow chart

The experimental study was a stepped-wedge cluster-randomized controlled trial. The trial was conducted between June 2018 and April 2019. Level II, III, and IV health centers that lacked access to a reliable, bright light source in the maternity ward were eligible for inclusion in the study. Facilities were randomized into one of two groups of 15 facilities to either receive the intervention in the first or second sequence. The focus of this analysis used data collected from the baseline (when no facility received the intervention) and the first follow up survey (6 weeks after the first 15 facilities had received the intervention) (Fig. 1). Within facilities, we interviewed all consenting health workers who were involved in labor and delivery. The interview consisted of both quantitative survey questions and qualitative open-ended questions. The purpose of the open-ended questions was to elicit narratives of the study context and intervention impact to facilitate interpretation of quantitative findings. Enumerators followed an interview guide to ask these open-ended questions and recorded health workers’ responses as field notes. Details of the trial design, including sampling methods, randomization, and sample size, have been previously published [25, 30].

A total of 15 female and 5 male enumerators conducted the health worker surveys. These enumerators had at least a certificate in Comprehensive Nursing or Midwifery and completed a 2-week training on research protocol, data collection methods, and human subjects research. With permission from the district and facility leaders, enumerators interviewed health workers at a private space using a questionnaire with both closed and open-ended questions. The research team comprised two researchers with PhDs in health policy and mixed-methods training (SR and WC), a researcher with a PhD in economics (JC), a researcher with a PhD in medicine (PW), and a researcher with a post-graduate diploma in monitoring and evaluation (BM). Three team members were female (JC, SR, and WC) and two were male (BM & PW).

### Sample size

Before the trial began, we calculated the minimum detectable effect size for health workers’ satisfaction with light and electricity to be 0.62, assuming a total of 3 health workers per facility across 30 facilities, a mean health worker satisfaction score of 2 (out of a 1–5 range), and an intra-cluster correlation (ICC) of 0.3. In practice, our observed sample size was about 2 health workers per facility, the baseline satisfaction score was 3.1, and the ICC was 0.2. Using these parameters, our ex post minimum detectable effect size for health worker satisfaction was 0.36.

### Measures

The pre-registered primary outcomes included health workers’ overall job satisfaction as well as their satisfaction with light and electricity. Job satisfaction was measured via an index and calculated as the mean of health workers’ responses to four statements about their motivation to work, how satisfied they are with their job, the morale level at their department, and their plans to stay at the same job, with higher scores indicating greater job satisfaction. Satisfaction with light and electricity is a binary variable equal to one if a health worker “agreed” or “strongly agreed” to both statements: “I am satisfied with the availability and brightness of light in this facility” and “I am satisfied with the availability of electricity in this facility.” Detailed definitions of quantitative outcomes are provided in Appendix Table 4.

For pre-registered secondary outcomes, we constructed an index to measure health worker’s experiences of the impact of lack of overhead light during nighttime deliveries in the past month. The index was calculated as the mean of 14 items that measured how often health workers conducted deliveries at night without overhead light; had to hold a torch (i.e., flashlight) in hand to see a patient; experienced lack of light that affected normal care provided; delayed care; feared to move around the facility; and were affected in ability to suture, find/use equipment, conduct examinations of the mother, provide emergency care, provide newborn care, monitor fetal heartrate, administer medication, clean up after delivery, and manage infection control. Responses were scored on a 1–5 Likert scale from “Never” to “Every nighttime delivery”, so that higher scores on the index indicated more frequent occurrence of negative impacts of lack of overhead light.

In addition to the pre-registered outcomes, we examined each of the indicators that constituted the 4-item job satisfaction index individually. We further examined outcomes that measured concepts closely related to health workers’ job satisfaction, such as job security, self-confidence, adequate support in terms of supplies and equipment, and workload. Responses to these measures were assessed on a 1–5 Likert scale to indicate levels of agreement, with higher scores indicating greater satisfaction.

As part of the open-ended questions, health workers were asked to comment on the impacts of lack of overhead light on their job, difficulties with using kerosene lanterns or candles, memorable situations where lack of lighting affected patient care, comparison between lack of lighting and other challenges at facilities, and their feelings about working without reliable light. After receiving the intervention, health workers were asked to comment on whether the intervention helped them to care for patients and what kind of challenges they encountered in using the intervention.

### Data analyses

First, we evaluated the effects of the intervention on health worker outcomes by analyzing quantitative data collected from health worker surveys. To supplement the quantitative findings, we analyzed qualitative data collected from the open-ended questions.

*Quantitative analysis* We used linear probability models that included facility fixed effects to estimate the impact of the intervention on primary and secondary outcomes at the level of health workers. Standard errors in all models were clustered at the facility level.

To assess the robustness of the models, we used alternative model specifications including non-linear models (logistic for binary outcomes and Poisson for count outcomes), facility random effects, inclusion of health worker control variables in regression models, and adjustment of standard errors using the wild cluster bootstrap method given the small number of facilities [33]. In addition, we assessed whether the results were driven by compositional changes in health workers after installation of the intervention by examining heath worker retention rates as a robustness check. Data were analyzed with Stata version 15.1 [34].

*Qualitative analyses* We used an inductive analytical approach to analyze the qualitative data [32]. One member of the research team began the qualitative analysis by reading the field notes in full until reaching a high level of familiarity with the content of the text. She organized enumerators’ field notes based on the open-ended questions, created initial codes by open coding the field notes, and coded the text to generate themes. A second member of the research team read the coded text and provided feedback. The two research team members iteratively analyzed the codes, coded the field notes, and finalized categories and themes. Throughout the process, we considered how our training, identity, and world view influenced our interpretation of the qualitative findings and consulted other research team members to draw on interdisciplinary insights. We did not present the qualitative results to health worker participants for comments, but we shared the findings with our in-country team who agreed that the findings reflected the reality in the clinical setting. Qualitative data were analyzed with Taguette version 0.10.1, a web-based text management and analysis software [35].

## Results

### Baseline characteristics and context

All eligible health workers agreed to participate in the health worker surveys (Fig. 1). At the baseline survey, 85 health workers participated in the health worker interview. At the follow-up survey, 54 health workers were interviewed. Common reasons for loss-to-follow-up included being transferred to another facility, having left the job, or being on maternity leave. The intervention assignment had no effect on health worker retention (Table 5).

Table 1 shows the baseline characteristics of health workers and facilities and tests for balance in these characteristics across facilities randomized to the intervention or control group. Characteristics of health workers retained vs. not retained in the follow-up survey are presented in Appendix Table 6. The majority of health workers were enrolled midwives or nurses (68%), followed by clinical or nursing officers (16%) and assistants or traditional birth attendants (15%). Almost all of them had post-secondary education (98%) with an average of 7.9 years of experience since obtaining their certificates. These health workers reported conducting an average of 17 deliveries in the past month. At the facility level, most of the 30 facilities were Level III Health Centers (73%) with an average patient volume of 33.6 per month. The intervention and control groups did not differ in health worker or facility characteristics. Health workers’ assessment of light and electricity and their job satisfaction levels were also similar at baseline.

**Table 1:** Baseline characteristics of health workers and health facilities

	Overall	Intervention	Control	*p*-value
Panel 1: Health Worker Characteristics				
Number of health workers	85	43	42	
Female	84 (99%)	43 (100%)	41 (98%)	0.31
Age, mean (SD)	34.2 (9.8)	33.4 (9.7)	35.1 (9.9)	0.43
Number of deliveries last month, mean (SD)	17.4 (15.4)	15.2 (12.1)	19.5 (18.0)	0.20
Education				
Only secondary	2 (2%)	2 (5%)	0 (0%)	0.27
Certificate	49 (58%)	26 (60%)	23 (55%)	
Diploma or Bachelor	34 (40%)	15 (35%)	19 (45%)	
Position				
Clinical/Nursing Officer	14 (16%)	7 (16%)	7 (17%)	0.11
Enrolled Midwife/Nurse	58 (68%)	26 (60%)	32 (76%)	
Assistant/Traditional Birth Attendants	13 (15%)	10 (23%)	3 (7%)	
Years of experience, mean (SD)	7.9 (8.4)	7.5 (8.1)	8.3 (8.7)	0.66
Satisfied with light and electricity	3 (4%)	1 (2%)	2 (5%)	0.54
Job satisfaction index, mean (SD)	3.1 (0.8)	3.1 (0.8)	3.1 (0.7)	0.99
Impact of lack of overhead light index, mean (SD)	3.2 (1.0)	3.2 (1.0)	3.1 (0.9)	0.64
Panel 2: Health Facility Characteristics				
Number of facilities	30	15	15	
Facility level				
Health Center II	5 (17%)	3 (20%)	2 (13%)	0.77
Health Center III	22 (73%)	11 (73%)	11 (73%)	
Health Center IV	3 (10%)	1 (7%)	2 (13%)	
Primary source of electricity				
None/lanterns	12 (40%)	6 (40%)	6 (40%)	0.89
Grid	11 (37%)	5 (33%)	6 (40%)	
Solar	7 (23%)	4 (27%)	3 (20%)	
Facility gov’t owned	28 (93%)	13 (87%)	15 (100%)	0.14
Monthly patient volume, mean (SD)	33.6 (17.2)	30.2 (18.1)	37.0 (16.1)	0.29

*MCH* maternal and child health, *SD* standard deviation. See Table 4 in the Appendix for definitions of outcomes

**Fig. 2:**
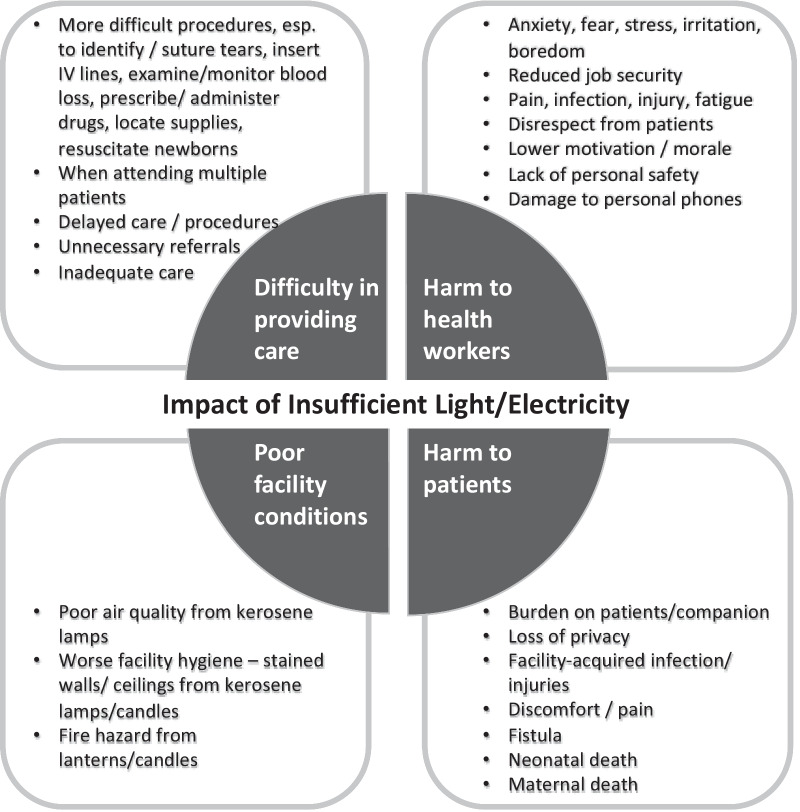
Impact of insufficient light or electricity based on qualitative open-ended questions with health workers

Qualitative data provided contextual information regarding what it was like to work in a maternity ward at night without sufficient light or electricity (Fig. 2 and Panel A of Table 7). Lack of light made it more difficult to provide care, affecting clinical procedures such as suturing, inserting IV lines, and monitoring blood loss. Sometimes these procedures were delayed until the morning or skipped altogether. Sometimes health workers referred out primigravida women by default because of the concern that they may not see clearly enough to suture potential tears appropriately. Lack of light or electricity also harmed health workers directly, as many felt anxious, fearful, or stressed to work at night without sufficient light and a few reported needlestick injuries. Health workers were also less motivated and felt a loss of dignity when they could not provide good care to patients. Most health workers used kerosene lamps at night, which stained the walls and ceilings, created a fire hazard, irritated the respiratory system, and only provided dim light that led to eyestrain. Other health workers reported needing to hold a torch or their own cell phone in the mouth, which made procedures harder to perform and led to damages when the phone was dropped by accident. Patients or their companions were asked to provide light or hold the torch during procedures, which eroded patient privacy and reduced community respect for health workers. Health workers also attributed a few cases of maternal and perinatal deaths to lack of light and electricity.

### Intervention effects on health worker’s satisfaction with light and electricity

Table 2 shows the main results from the regression analyses. The intervention did not have a significant effect on health worker’s satisfaction with light and electricity together. However, looking at light and electricity separately, the intervention increased health workers’ satisfaction with light by 76 percentage points [95% confidence interval (CI): 61–92 percentage points], an almost 20-fold increase from the control mean of 4%. In comparison, the intervention had no effect on health workers’ satisfaction with electricity. Restricting the sample to health workers present at both surveys (Panel B of Table 2) or analyzing the outcomes as continuous instead of binary (Table 8) did not change the results.

**Table 2:** Regression estimates of intervention impact on health worker outcomes

	(1)	(2)	(3)	(4)	(5)
	Satisfied with light and electricity	Satisfied with light	Satisfied with electricity	Impact of lack of overhead light index	Job satisfaction index
Panel A: All health workers presented at baseline					
Solar Suitcase	0.19	0.76***	0.19	− 1.96***	0.30**
	[− 0.05, 0.43]	[0.61, 0.92]	[− 0.05, 0.43]	[− 2.63, − 1.29]	[0.08, 0.51]
Control mean	0.04	0.04	0.04	3.28	3.17
Observations	139	139	139	136	139
Health workers	85	85	85	85	85
Panel B: Health workers presented at both baseline and follow-up surveys					
Solar Suitcase	0.20	0.76***	0.20	− 1.98***	0.33**
	[− 0.07, 0.47]	[0.62, 0.90]	[− 0.07, 0.47]	[− 2.71, − 1.25]	[0.11, 0.55]
Control mean	0.04	0.04	0.04	3.38	3.19
Observations	108	108	108	105	108
Health workers	54	54	54	54	54

Linear models that use data from both baseline and follow-up surveys and include facility fixed effects. Results show point estimate and 95% confidence interval. Standard errors are clustered at facility level. Satisfied with light and electricity is equal to 1 if health worker strongly agrees or agrees with both (1) I am satisfied with the availability and brightness of light in this facility and (2) I am satisfied with the availability of electricity in this facility. Impact of lack of overhead light index is an index of 14 items measuring health workers’ assessment of impact of lack of overhead light on their ability to conduct job tasks, including how often health worker: conducted deliveries without overhead light; had to hold torch in hand/mouth to see patient; experienced lack of light that affected normal care provided; delayed care; feared to move around facility; was affected in ability to suture, find/use equipment, conduct examinations of mother, provide emergency care, provide newborn care, monitor fetal heartrate, administer medication, clean up after delivery, manage infection control. This is reversely coded so that higher score indicates more frequent negative impact. Job satisfaction index is the mean score of four statements on a 1–5 scale: (1) These days, I feel motivated to work as hard as I can. (2) Overall, I am satisfied with my job. (3) Overall, the morale level at my department is good (4) I plan on staying at this position for the next year. *p < 0.05, **p < 0.01, ***p < 0.001

Similarly, the intervention significantly reduced health worker’s experience of negative impacts of lack of overhead light, lowering the index score by 1.96 points (95% CI: − 2.63 to − 1.29) from a control mean of 3.28 points. This can be interpreted as a change from lack of overhead light affecting workers during some nighttime deliveries before the intervention to almost never affecting workers during nighttime deliveries after the intervention.

Qualitative data supplemented these results by showing how the intervention made many clinical and administrative procedures easier to perform, such as conducting episiotomy, suturing tears, and removing the placenta (Table 3). In their response to the open-ended questions, health workers also provided many examples of how the headlamp, overhead light, and fetal Doppler from the intervention reduced unnecessary referrals and delays in care, enhanced health workers’ personal security, and created a better work environment overall.

**Table 3:** Results of thematic analysis of qualitative open-ended questions on Solar Suitcase

Themes	Codes	Representative examples from field notes
*In response to the question:* *Please comment more about any of the items you identified, such as one or more memorable situations when the Solar Suitcase LED lights, headlamps, phone charging ports and/or fetal Doppler were able to help you or other staff at the health facility in caring for a patient*		
Assist in procedures	Doppler, headlamp, overhead light	*One day [the health worker] was using a fetoscope on a mother in labor and could not hear the heart beat as she has a hearing problem, but with the Doppler she was able to get the heart rate and it was normal* *Midwife received a mother late about 1 month ago who was a multigravida and in second stage of delivery. Mother got a third-degree tear. [The midwife] was able to see very well while suturing with the aid of the headlamp and light from the Solar Suitcase. She praised the headlamp having a bright focused light which made the procedure to be done easily without difficulty* *[The health worker] gave an episiotomy with help of the overhead light and sutured it after still using the same head light.it really helped as compared to those days when she had to hold a torch in her mouth or call the attendant to help her hold*
Avoid unnecessary referral	Doppler, headlamp, suitcase	*There was a time when [the health worker] failed to get the fetal heart rate of a mother who she almost referred out to hospital due to IUFD [intrauterine fetal demise]. Then she remembered the fetal Doppler which she used and it helped her get a clear fetal heart rate of the baby successfully, kept the mother with her until the mother delivered a live baby* *One night the nursing assistant had a delivery after which she came and called [the health worker]that the mother had got a third-degree tear and wanted to refer the mother, so [the health worker] got the headlamp and examined closely only to realize that the tear had just extended near the anus. [The health worker was then able to repair it with the headlamp on* *Midwife reported to have received a mother with retained placenta at around 01:00 h when they had just received the Solar Suitcase, and because the Solar Suitcase was providing very bright light for manual removal of the placenta to be done, she didn’t refer the patient as it was the trend when they hadn’t received the Solar Suitcase*
Reduce delay in diagnose, treatment, or referral	Doppler, overhead lamp	*Last month Antenatal mother came in third trimester and [the health worker] examined her with fetoscope; fetal heart was not clear, so [the health worker] used fetal Doppler to find out that fetal heart rate was 173 b/m. [The health worker] suspected fetal distress then immediately referred the mother to a bigger facility for further evaluation* *One day [the health worker] had a mother he delivered well. Both the mother and the baby were transferred to postnatal ward in a good condition, but after that the cord started oozing some blood. It was only because of the solar led lights that he was able to detect it early enough and retied the cord again*
Enhanced personal security	Headlamp	*One night a mother came at night and [the health worker] was called from the staff quarters to come and examine the mother. [The health worker] was holding the headlamp to light the way since the staff houses are about 100 m away from the labor ward. [The health worker] saw a medium sized black snake in the grass near the path. [The health worker] called the night watchman and they killed it*
Reduce stress/ create better work environment	Overhead lamp	*[The facility] had a solar which was unreliable and could go off any time. Now the lights are reliable; [health workers] deliver a mother when they are relaxed. No unnecessary augmenting because [health workers]are fearing the lights to go off before mother gives birth*
Increase patient satisfaction	Doppler	*The fetal Doppler quickens work most especially when carrying out antenatal exams. There was a day when mothers were many but they were all worked on in a very short time and that day they were very happy*
Enable mothers to stay postpartum	Overhead lamp	*Solar Suitcase has been able to provide light in postnatal and labor suite now when it’s 20:00 pm. After delivery, mothers stay till morning because there is adequate lighting*
Emergency communication	Phone charger	*[Health workers] charged the phone to call a doctor for C-section as the facility had taken almost the whole week without power*
Reduced cost	Phone charger	*[The health worker] always keeps her phone fully charged because Solar Suitcase is always available. She is able to consult with a midwife if the midwife is away about patient care in maternity. Before the Solar Suitcase came, they used to take phones to a local trading center for charging and would pay money*

### Intervention effects on job satisfaction

The intervention significantly increased health workers’ job satisfaction by 0.30 points (95% CI: 0.08–0.51), an increase of about 10% from the control mean (Table 2). Restricting the sample to health workers present at both surveys did not change the results (Panel B of Table 2). Regarding the individual items included in the job satisfaction index, the intervention increased health workers’ reported motivation to work hard by 0.6 points (95% CI: 0.02–1.17) from a control mean of 3.18, but had almost no effect on health workers’ job satisfaction when assessed as a single question, nor on their morale or intention to stay (Table 8).

The qualitative data provided insights that may explain the lack of stronger intervention effects on health workers’ job satisfaction (Panel B of Table 6). First, health workers mentioned a few problems related to using the Solar Suitcase effectively, such as the short battery span of the overhead light, no refill of ultrasound gel for the Doppler, and lack of training on how to use the Solar Suitcase for new staff who joined after the original installation. Based on the quantitative data, 24% of health workers recruited in the follow-up survey were not fully trained on how to use the Solar Suitcase. Second, health workers faced many other challenges at the facility in addition to insufficient light, such as lack of supplies, space, clean water, security, and accommodation. Moreover, poor community relationships and understaffing may have lowered the feeling of fulfillment health workers could derive from their job despite better infrastructure.

**Fig. 3:**
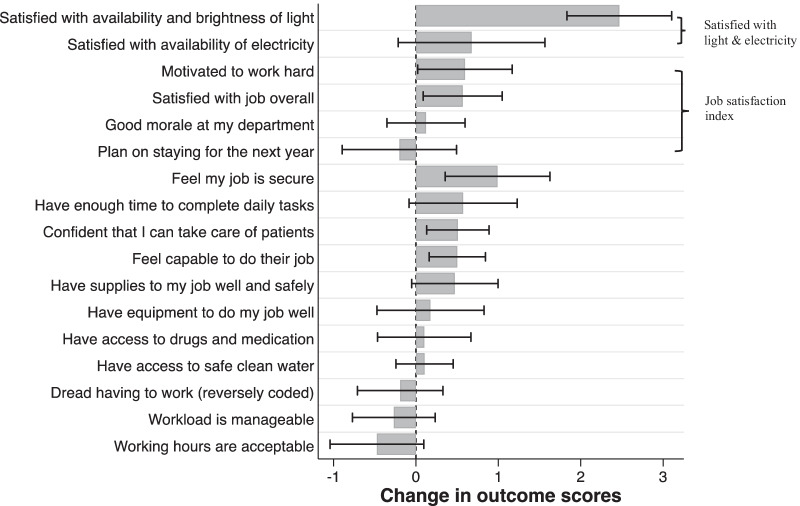
Impact of intervention on individual items related to job satisfaction. Linear regression results show point estimates and 95% confidence interval. Standard errors are clustered at facility level. Sample consists of all health workers present at the baseline survey. Health workers rated to what extent they agreed with each statement on a 1–5 scale, with 1 being “strongly disagree” and 5 “strongly agree”.

In addition to the primary outcome on job satisfaction, the intervention had significant positive effects on health workers’ sense of job security, confidence in their capacity to take care of patients, and capability to do their job (Fig. 3). Based on the qualitative data (Table 3), midwives worried about losing their job because they could not do it well without adequate light, which may explain the effect on job security. Health workers reported that the fetal Doppler that came with the intervention made detecting the fetal heart rate much faster and easier than a fetoscope, which improved the timeliness of care and increased patient satisfaction. Health workers also reported that the intervention provided lighting necessary to monitor patients and detect complications, which reduced delay in diagnosis and treatment. These qualitative examples illustrated how the intervention may have strengthened health workers’ confidence in providing good patient care.

In our robustness checks, we found that using alternative model specifications produced generally similar results (Table 9). The exceptions were for satisfaction with light and electricity and the job satisfaction index in non-linear models. In the logistic model, the odds of being satisfied with light and electricity was significantly higher for the intervention group. In the Poisson model, the intervention had a positive but not significant effect on the incidence rate ratio for the job satisfaction index. Including facility random effects or adjusting standard errors using the wild bootstrap method did not change the results presented in Table 2. Adding health worker controls to the regression models did not change the results (Table 10).

## Discussion

Using a mixed-methods approach, this study examined whether and how an infrastructure-improvement intervention affected the job satisfaction of health workers who provided maternal and newborn care in rural Uganda. The results show that the Solar Suitcase installed in maternity facilities improved health workers’ satisfaction with light but not with electricity, reduced health worker’s experiences of negative impacts of lack of overhead light during night-time deliveries, and had a modest effect on health workers’ job satisfaction. The intervention addressed most problems posed by insufficient light in maternity facilities and strengthened health workers’ sense of job security and confidence in providing high-quality care. However, a range of challenges may have prevented the Solar Suitcase from having a larger impact on the job satisfaction of health workers who often faced many prominent barriers in a resource-constrained setting.

The installation of the Solar Suitcase increased the proportion of health workers who were satisfied with light from 4% to more than 80%. The intervention also reduced how often lack of overhead light negatively affected health workers’ ability to provide maternity and newborn care, essentially reducing the impacts from affecting some nighttime deliveries to nearly never affecting deliveries. These large impacts on subjective measures of light and electricity echo our findings previously that the Solar Suitcase was well-adopted and increased the brightness and adequacy of lighting measured by enumerators and light sensors [36]. Given these large effects, future research could explore the Solar Suitcase’s application in other clinical domains. However, the intervention did not significantly increase health worker’s satisfaction with electricity, probably because electric power derived from the Suitcase was limited to charging mobile phones and small medical devices, as opposed to, for example, powering a refrigerator to store blood products or powering the whole facility. It is also possible health workers interpreted “electricity” to mean availability of the electric grid, which the Suitcase did not affect.

Results from the study also illustrated how improvement in health infrastructure could enhance health workers’ job satisfaction, as suggested by previous research in LMIC [24, 37, 38]. The intervention increased health workers’ score on the job satisfaction index by about ten percent. Qualitative evidence suggested that better lighting and electricity improved work environment, living conditions, and personal security, which are especially important to female health workers in rural areas [39]. In addition, sufficient light may also have signaled higher quality of care to the community, earned more respect for health workers, and made their work more rewarding, which all contribute to greater job satisfaction [15, 39]. Given the importance of health workers’ motivation to successful guideline implementation in obstetric care in LMICs and the limited existing research on effective approaches to motivate health workers [9, 40], our study contributes to the literature by generating rigorous and contextual evidence for cross-sectional investment in health infrastructure as a policy option for human resources for health. [7]

However, the intervention’s impact on job satisfaction was only modest and not always significant in our sensitivity analyses. One explanation could be that while the intervention provides bright and reliable light, it is limited in that it is only installed within the maternity ward, leaving other facility areas unlit, and may not meet all of a facility’s energy needs. In addition, factors other than light or electricity, such as financial incentives, career development, or management issues [37], also play a significant role in health workers’ job satisfaction in our study setting. Qualitative data also underlined other facility challenges important to health workers, such as lack of medical supplies, equipment, space, security, and training, which are common challenges for health workers in resource-constrained settings [9, 38, 41]. Consistent with previous evidence [9, 40, 42, 43], our findings highlight the necessity of reliable light for health facilities to improve quality of maternal and newborn care while pointing toward the importance of comprehensive interventions tailored to address multiple barriers in a health system.

This study has several limitations. First, we were unable to identify or use a validated scale to measure job satisfaction of health workers that provide maternal and neonatal care in our study setting. However, we drew from existing measurement developed in similar settings, covered key aspects that determine job satisfaction for health workers in general, and supplemented quantitative surveys with qualitative data [44–46]. Second, there is a possibility of interviewer bias as health workers might have felt compelled to report higher job satisfaction after receiving the Solar Suitcase. However, the main items included in the job satisfaction index were not directly related to light or electricity. In addition, qualitative data provided ample examples of specific patient cases where the Solar Suitcase was critical to maternity care. Third, we were not able to conduct a thorough qualitative investigation that analyzed fully-transcribed in-depth interviews with health workers. Future research could conduct a stand-alone qualitative inquiry for a deeper understanding of the intervention’s impact on health workers. Fourth, the study had a small sample size and due to high turnover, we were only able to re-interview 64% of health workers in the follow-up survey, which might explain the lack of effects on individual job satisfaction outcomes. However, our power analysis showed that the study was sufficiently powered on the main job satisfaction index and health workers lost at the follow-up survey were not different from the remaining ones based on observed characteristics. Finally, our study assessed the effects of the intervention within a relatively short time period. A longer follow-up survey could reveal whether and how a one-time investment on health infrastructure could have sustained impact on health workers and maternity care.

## Conclusions

Maintaining a motivated health workforce is essential to quality of maternal and neonatal care, but evidence on effective strategies to improve health workers’ job satisfaction in resource-constrained settings is limited. We find that installation of a solar electric system addresses the challenges posed by insufficient light in maternity facilities, enhances health workers’ ability to provide maternal and neonatal care, and may be an important determinant of health workers’ job satisfaction. Policy makers should consider greater investment in health infrastructure to strengthen human resources for health and to improve maternal and newborn care, as part of multifaceted, systemic policy changes for health system strengthening.

## Data Availability

The data sets during and/or analyzed during the current study available from the corresponding author on reasonable request.

## Appendix

See Tables 4, 5, 6, 7, 8, 9, 10.

**Table 4:** Outcome measurements and definitions

Outcome	Type (No. of Items)	Definition
Pre-specified primary outcomes		
Satisfied with light and electricity	Binary (2)	Equal to 1 if strongly agree or agree with both statements: (1) I am satisfied with the availability and brightness of light in this facility. (2) I am satisfied with the availability of electricity in this facility
Job satisfaction index	Continuous (4)	Mean score (1–5) of response to four statements: (1) These days, I feel motivated to work as hard as I can. (2) Overall, I am satisfied with my job. (3) Overall, the morale level at my department is good (4) I plan on staying at this position for the next year
Pre-specified secondary outcomes		
Impact of lack of overhead light index	Continuous (14)	Index of items measuring how lack of overhead light in facilities affected health workers’ ability to perform job functions, including how often health worker: conducted deliveries without overhead light; had to hold torch in hand to see patient; experienced lack of light that affected normal care provided; delayed care; feared to move around facility; was affected in ability to suture, find/use equipment, conduct examinations of mother, provide emergency care, provide newborn care, monitor fetal heartrate, administer medication, clean up after delivery, manage infection control. Response to items scored (1–5) on following scale: 1. Every delivery, 2. Most, 3. Some, 4. Few, or 5. Never. Overall score is the average of 14 items, yielding a range of 1–5. We reverse-coded the index so that higher overall score indicates more frequent occurrence of negative impact of lack of overhead light
Other outcomes in the main results		
Satisfied with light	Binary	Equal to 1 if strongly agree or agree with: I am satisfied with the availability and brightness of light in this facility
Satisfied with electricity	Binary	Equal to 1 if strongly agree or agree with: I am satisfied with the availability of electricity in this facility

**Table 5:** Effects of intervention assignment on retention

	Retained
Assigned to intervention at baseline	− 0.04
	[− 0.23, 0.16]
Control mean	0.84
Observations	88

A health worker was considered “retained” if she was (1) either interviewed for the health worker survey or observed in delivery observations at baseline and (2) re-interviewed or re-observed in the follow-up data collection round. Models included facility fixed effects. Results show point estimate and 95% confidence interval. Standard errors are clustered at facility level

**p* < 0.05, ***p* < 0.01, ****p* < 0.001

**Table 6:** Characteristics of health workers for subgroup analyses

	Retained	Not retained	*p*-value
Number of health workers	54	31	
Female	54 (100%)	30 (97%)	0.18
Age, mean (SD)	34.4 (9.4)	33.8 (10.6)	0.79
Number of deliveries last month, mean (SD)	18.7 (15.9)	15.0 (14.4)	0.30
Education			
Only secondary	0 (0%)	2 (6%)	0.15
Certificate	31 (57%)	18 (58%)	
Diploma or Bachelor	23 (43%)	11 (35%)	
Position			
Clinical/Nursing Officer	9 (17%)	5 (16%)	0.026
Enrolled Midwife/Nurse	41 (76%)	17 (55%)	
Assistant/Traditional Birth Attendants	4 (7%)	9 (29%)	
Years of experience, mean (SD)	8.4 (8.8)	7.0 (7.6)	0.48
Satisfied with light and electricity	2 (4%)	1 (3%)	0.91
Job satisfaction index, mean (SD)	3.1 (0.7)	3.1 (0.8)	0.94
Impact of lack of overhead light index, mean (SD)	3.3 (1.0)	3.0 (1.0)	0.25

*SD* standard deviation

**Table 7:** Results of thematic analysis of qualitative open-ended questions on impact of insufficient light or power

Themes	Codes	Representative examples from field notes
Panel A: Impact of Insufficient Light or Power		
* In response to the questions:* *Please comment on the aspects of your job where lack of overhead light impacts you the most* *Please comment on any problems or difficulties resulting from your use of kerosene lanterns or candles such as: personal injuries, candle wax burns, problems with eyes from smoke, problems with breathing from smoke, burn marks on wall, *etc*.?* *Please describe in detail a memorable situation when you were trying to care for a patient, and you did not have sufficient lighting*		
Difficulty in providing care	More difficult procedures	*Suturing an episiotomy or tears is difficult whereby the midwife has to hold a torch in the mouth and sometimes pierces herself in the process* *Another point is when assessing to confirm PPH, it is difficult to distinguish fresh blood and the non-fresh because of lack of adequate light* *Used instruments can only be cleaned in the mornings. It’s impossible to clean the floor in dim light* *One time an infant was brought with severe malaria with convulsions and [the health workers] used the headlamps to pass an intravenous line and gave emergency treatment and he got better* *During resuscitation of the baby, it’s hard to see the chest movements if there’s no overhead light and ruling which color of the skin is the baby* *It was March and the midwife was giving routine care to a baby by cutting the baby’s cord and a torch which was put in her mouth fell down and stopped working. She tried to repair it and it refused so she had to cut the cord in darkness. If there was good lighting, she would have cut it without any delay*
	When attending multiple patients	*It was one night when [the health worker] was conducting a delivery and she had positioned the torch in a place where it was focusing light very well to this mother on the delivery bed, meanwhile [the health worker] was called that another mother had come and also in second stage so she just rushed to go and help the other who seemed to be pushing at that time. However, [the health worker] forgot the torch in the delivery room and couldn’t carry it because she had gloved already. After reaching there, she couldn’t see very well and rushed back to get the torch. However, when [the health worker] came back, she found the baby was down on the floor. [The health worker] tried to do immediate care to the newborn and third stage but it would be different if she had adequate light: the baby wouldn’t have fallen on the floor because [the health worker] was around when the delivery began, but all changed when [the health worker] ran for the torch*
	Delayed care/procedures	*It was a night delivery and the mother was nearing second stage when the lantern went off. The paraffin had been used up and [the health worker] didn’t realize that earlier to prepare for it. [The health worker] conducted the delivery without any light source since it was very late. [The health worker] did not assess for tears till morning, and yet the woman had sustained one, which [the health worker] repaired the following morning*
	Unnecessary referrals	*Most prime gravida are referred due to fear that they may get tears because inadequate light would lead to poor suturing*
	Inadequate care	*HIV positive mother got a tear. Because there was no light, she was left unsutured. If there were light, mother would be sutured*
Harm to health Worker	Anxiety, fear, stress, feeling bad/irritated, boredom	*In case there’s no light during these scenarios it makes the health worker panic, nervous and feel like if something bad happens her job will be gone* *So bad and disturbing, whenever night comes, [the health worker] thinks of work without power and prays that no mother comes in the night*
	Reduced job security	*It’s a very bad situation. If it happens, midwife fears to lose her job in case a fatal situation happens* *It has made some mothers not to come to the facility with fear of delivering in the dark. The midwife claimed that lack of reliable lighting has led to low generation of income to run the facility*
	Physical pain, infection, injury, tiredness	*Giving treatment, calculating doses, and checking expiry dates are difficult because dim light makes the midwife’s eyes develop pain at night* *During night deliveries, when [the health worker] has to use a phone torch to conduct a delivery and the torch is also dim, [the health worker] can end up suturing herself*
	Disrespect from patients	*Very bad. It makes every care provided to the mother to be meaningless and vanishes the respect the mothers and the companion can give to the midwife.*
	Lower motivation/morale	*It’s a terrible moment which makes [the health worker] feel like leave her job cause it puts the baby’s and mother’s life in danger as well as spoiling her career and dignity* *It’s a condition that the health worker doesn’t like to work in. It makes her feel like she’s in a wrong profession especially at night*
	Lack of personal safety	*One time, after delivery as the midwife was taking the placenta to the placenta pit, she almost stepped on a snake without seeing it. This is because there is no light around that area*
	Damage to personal phones	*At 3am mother came in second stage and [the health worker] helped the mother to deliver with the help of a torch in the mouth so as to see, but along the way of cleaning, the phone fell into liquor and got damaged. It was difficult to replace it and yet if light were sufficient, it wouldn’t have happened that way*
Poor facility conditions	Worse facility hygiene	*Soot makes the walls look nasty, giving the cleaning department more work. This would not have happened if lamps weren’t being used*
	Fire hazard from lanterns/candles	*There can be risks of fire outbreak when using candles because they are the main source of light used in case electricity is off*
Harm to patients	Burden on patients/companion	*In January, a mother was laboring when the husband fainted in the labor ward. Husband was told to help hold the torch for the delivery. He would have just waited for the baby in the postnatal ward*
	Loss of privacy	*[The health worker] was conducting a delivery of a precious baby and was using a lantern. She had to call another person to help her position the lamp wherever she wanted so as to finish the delivery as well as to resuscitate but the privacy was compromised because the person was not even a health worker*
	Facility-acquired infection/injuries	*The soot leads to respiratory infections especially for babies who are born under the dark and stay for 2 days in the department*
	Discomfort/pain	*It happened around December 2016. The midwife was suturing a vaginal tear when grid light was off and the solar at the facility wasn’t reliable as it produces dim light. [The health worker] focused a torch to the mother which was in her mouth. [The midwife] said that the suturing took a long time and the local anesthetic drug wore off. The mother was in pain. The midwife pierced herself several times. If [the midwife] had good light, the suturing process would have been quick*
	Fistula	*Due to the poor reliability and brightness of light used when the overhead light is absent, it puts the health worker at risk of possible cross infection. Poor suturing not in the respective layers might make the mother develop fistula*
	Neonatal death	*Mother came in second stage with raptured membranes. 1*^*st*^* baby was delivered with Apgar score of 5 at 1*^*st*^* minute and at 5 min 0/10 (1.3 kg). Second baby came at 21:39 h with Apgar score of 0 (0.6 kg) at 1 min. Third baby at 22:00 with Apgar score of 6/10 at 1 min (1.3 kg) and was referred but also passed on after 2 days in Hospital. These babies would have been resuscitated well and if there were light and electricity, their lives would have been saved*
	Maternal death	*There was a mother who came with a preterm pregnancy and she was already in second stage, so [the health worker] conducted the delivery, but because there was no sufficient lighting, [the health worker] didn’t notice the mother was anemic. [The health worker] referred the mother the next morning to [X] hospital but unfortunately, she died on the way. The story would have been different: if [the health worker] had adequate light, she would have referred her early*
Panel B: Reasons Solar Suitcase Might Not Be Enough		
* In response to the questions:* *Please comment on any problems or difficulties you’ve had with using the Solar Suitcase or any of its components* *How does lack of overhead light compare to other problems at this facility? Is it the most important problem, or are there other areas that you feel are more important to address?*		
Problems with Solar Suitcase	Overhead LED light—does not last	*When all 4 bulbs are switched on, sometimes when it has rained, lights go out very early in the morning at around 4:00am. Thus, if a mother comes that time, she delivers in the dim electricity. It has occurred once since it was installed*
	Overhead LED light—not bright enough	*One night [the health worker] had a mother with a second-degree tear, yet the light from the solar was not enough and the mother was developing PPH [postpartum hemorrhage]. [The health worker] had to use the headlamp to repair the tear and then the bleeding also reduced*
	Doppler—unstable reading	*Sometimes the fetal Doppler keeps disturbing [the health worker] by giving high and low reading some time*
	Doppler—gel running out	*The only problem is when the gel gets done but other things are well and fine*
	Doppler—charging problems	*One day [the health worker] had a mother and couldn’t use a fetal scope to check for fetal heart rate. The fetal Doppler also failed because it had not charged because the charger was faulty*
	Lack of training/	*Midwife wasn’t trained on how to use it and admitted to have problems with charging the fetal Doppler, headlamp and the batteries separately, interpreting the charger lighting to detect any problems*
	Infrequent use	*[The health worker] has hardly used the Solar Suitcase. She has only used it like once*
Other challenges	Lack of medical supplies/equipment	*It’s not the only problem of the facility; there are other problems affecting the facility, including lack of mama kits to give out during antenatal visits or during delivery, lack of a resuscitation table, delivery bed, or equipment used in delivery (such as suture holders, episiotomy scissors, malfunctioning suction bulbs), lack of an incinerator or autoclave to sterilize equipment, low funds to purchase drugs (e.g., oxytocin and antibiotics) as the government supplies only class C drugs which are not the most needed at the facility*
	Lack of space	*The maternity ward is very small with one delivery bed and one postnatal bed. Maybe those can be addressed first*
	Lack of clean water	*[The health worker] stated that accessing water at the facility is her first priority and light problems would come second since at times they even have to postpone cleaning after delivery because of lack of water, especially in the dry season*
	Poor transportation/road conditions	*Mothers came in second stage due to long distances and poor roads, which make them delay care. In addition, poverty in the community makes mothers go to traditional birth attendants and when it complicates, mothers are referred sometimes in second stage*
	Inadequate security	*Lack of security personnel and fence for the health facility as well as staff quarters*
	Inadequate accommodation	*The health worker feels the issue of accommodation should also be addressed because the staff quarters are not enough. Some of them improvised by sleeping in some rooms on the medical wing*
	Poor community relationship	*Harsh community habits towards health workers, especially if there’s a stockout of drugs or equipment*
	Under staffing/heavy workload	*Work overload, especially during clinic days for family planning, antenatal, HIV *etc*. There are usually only 2 midwives on duty*
	Improper waste disposal	*Waste segregation bins are not enough and placenta pit is not in good condition*
	Patient poverty	*Poverty has affected a lot of the health sector as some mothers are unable to provide baby clothes (babies are wrapped in cotton or men’s shirts). Some even fail to get a meal after birth*
	Late pay	*The midwife said that she felt demotivated to work because she hasn’t received her salary for 4 months*
	Inadequate training/supervision	*Inadequate continuous medical evaluation from supervisors. Poor supply of equipment. No in-service trainings*

**Table 8:** Estimates of intervention impact on individual items in the satisfaction indices

	(1)	(2)	(3)	(4)	(5)	(6)	(7)	(8)
	Satisfied with light	Satisfied with electricity	Light and electricity satisfaction Index	Motivated to work hard	Satisfied with job	Good morale	Plan on staying next year	Job satisfaction Index
Panel A: All health workers presented at baseline								
Solar Suitcase	2.47***	0.68	1.46***	0.60*	0.57*	0.12	− 0.20	0.30**
	[1.83, 3.10]	[− 0.21, 1.57]	[0.76, 2.16]	[0.02, 1.17]	[0.09, 1.05]	[− 0.35, 0.60]	[− 0.90, 0.49]	[0.08, 0.51]
Control mean	1.51	1.55	1.54	3.18	3.40	3.37	2.74	3.17
Observations	138	129	129	139	138	138	138	139
Health workers	84	82	82	85	85	85	85	85
Panel B: Health workers presented at both baseline and follow-up								
Solar Suitcase	2.40***	0.53	1.31**	0.56*	0.49	0.29	− 0.08	0.33**
	[1.69, 3.11]	[− 0.49, 1.56]	[0.51, 2.12]	[0.05, 1.07]	[− 0.10, 1.09]	[− 0.09, 0.67]	[− 0.86, 0.69]	[0.11, 0.55]
Control mean	1.53	1.56	1.55	3.20	3.49	3.34	2.73	3.19
Observations	108	101	101	108	107	107	107	108
Health workers	54	54	54	54	54	54	54	54

Linear models that use data from both baseline and follow-up surveys and include facility fixed effects. Results show point estimate and 95% confidence interval. Standard errors are clustered at facility level. Light and electricity satisfaction index is the mean score of two statements on a 1–5 scale: (1) I am satisfied with the availability and brightness of light in this facility. (2) I am satisfied with the availability of electricity in this facility. Job satisfaction index is the mean score of four statements on a 1–5 scale: (1) These days, I feel motivated to work as hard as I can. (2) Overall, I am satisfied with my job. (3) Overall, the morale level at my department is good (4) I plan on staying at this position for the next year

**p* < 0.05, ***p* < 0.01, ****p* < 0.001

**Table 9:** Estimates of intervention impact using alternative model specifications

	Model 1	Model 2	Model 3	Model 4
Satisfaction with light and electricity	8.68^*^	0.19	0.19	0.19
	[1.30, 58.16]	[− 0.03, 0.40]	[− 0.03, 0.40]	[− 0.00, 0.46]
Observations	139	139	139	139
Control mean	0.04	0.04	0.04	0.04
Satisfaction with light	87.20***	0.75***	0.76***	0.76***
	[19.75, 384.98]	[0.62, 0.88]	[0.63, 0.90]	[0.60, 0.91]
Observations	139	139	139	139
Control mean	0.05	0.04	0.04	0.04
Satisfaction with electricity	6.88	0.18	0.19	0.19
	[0.89, 53.01]	[− 0.03, 0.39]	[− 0.03, 0.40]	[− 0.00, 0.46]
Observations	139	139	139	139
Control mean	0.05	0.04	0.04	0.04
Impact of lack of overhead light index	0.40***	− 1.96***	− 1.96***	− 1.96***
	[0.34, − 0.46]	[− 2.46, − 1.47]	[− 2.56, − 1.37]	[− 2.75, − 1.33]
Observations	136	136	136	136
Control mean	3.28	3.28	3.28	3.28
Job satisfaction index	1.06	0.24^*^	0.30^**^	0.30^*^
	[0.95, 1.17]	[0.04, 0.45]	[0.11, 0.49]	[0.09, 0.50]
Observations	139	139	139	139
Control mean	3.17	3.17	3.17	3.17
Regression model	Logistic/Poisson	Linear	Linear	Linear
Facility fixed/random effects	None	Random	Fixed	Fixed
Bootstrapped standard errors	No	No	No	Yes

Linear models report coefficients. Logistic models report odds ratios for binary variables. Poisson models report incident rates for count variables. Standard errors are clustered at facility level. Bootstrapped standard errors are calculated using the wild cluster bootstrap method. Satisfied with light and electricity is equal to 1 if health worker strongly agrees or agrees with both (1) I am satisfied with the availability and brightness of light in this facility and (2) I am satisfied with the availability of electricity in this facility. Impact of lack of overhead light index is an index of 14 items measuring health workers’ assessment of impact of lack of overhead light on their ability to conduct job tasks, including how often health worker: conducted deliveries without overhead light; had to hold torch in hand/mouth to see patient; experienced lack of light that affected normal care provided; delayed care; feared to move around facility; was affected in ability to suture, find/use equipment, conduct examinations of mother, provide emergency care, provide newborn care, monitor fetal heartrate, administer medication, clean up after delivery, manage infection control. This is reversely coded so that higher score indicates more frequent negative impact. Job satisfaction index is the mean score of four statements on a 1–5 scale: (1) These days, I feel motivated to work as hard as I can. (2) Overall, I am satisfied with my job. (3) Overall, the morale level at my department is good (4) I plan on staying at this position for the next year

**p* < 0.05, ***p* < 0.01, ****p* < 0.001

**Table 10:** Estimates of intervention impact on health worker outcomes—controlling for health worker characteristics (age, position, deliveries last month, and education)

	(1)	(2)	(3)	(4)	(5)
	Satisfied with light and electricity	Satisfied with light	Satisfied with electricity	Impact of lack of overhead light index	Job satisfaction index
Panel A: All health workers presented at baseline					
Solar Suitcase	0.19	0.75***	0.19	− 1.97***	0.31**
	[− 0.06, 0.44]	[0.60, 0.91]	[− 0.06, 0.44]	[− 2.56, − 1.37]	[0.09, 0.53]
Control mean	0.04	0.04	0.04	3.29	3.18
Observations	137	137	137	135	137
Health workers	84	84	84	84	84
Panel B: Health workers presented at both baseline and follow-up surveys					
Solar Suitcase	0.20	0.75***	0.20	− 1.97***	0.31*
	[− 0.08, 0.47]	[0.60, 0.91]	[− 0.08, 0.47]	[− 2.63, − 1.31]	[0.06, 0.56]
Control mean	107	107	107	3.38	3.20
Observations	54	54	54	105	107
Health workers	0.04	0.04	0.04	54	54

Linear models that use data from both baseline and follow-up surveys and include facility fixed effects. Results show point estimate and 95% confidence interval. Standard errors are clustered at facility level. Satisfied with light and electricity is equal to 1 if health worker strongly agrees with both (1) I am satisfied with the availability and brightness of light in this facility and (2) I am satisfied with the availability of electricity in this facility. Impact of lack of overhead light index is an index of 14 items measuring health workers’ assessment of impact of blackouts on their ability to conduct job, including how often health worker: conducted deliveries without overhead light; had to hold torch in hand to see patient; experienced lack of light that affected normal care provided; delayed care; feared to move around facility; was affected in ability to suture, find/use equipment, conduct examinations of mother, provide emergency care, provide newborn care, monitor fetal heartrate, administer medication, clean up after delivery, manage infection control. This is reversely coded so that higher score indicates more frequent negative impact. Job satisfaction index is the mean score on a 1–5 scale to four questions (1) These days, I feel motivated to work as hard as I can. (2) Overall, I am satisfied with my job. (3) Overall, the morale level at my department is good 4) I plan on staying at this position for the next year. See Table 1 in the Appendix for definitions of outcomes. **p* < 0.05, ***p* < 0.01, ****p* < 0.001
